# Population-based analyses of *Giardia duodenalis* is consistent with the clonal assemblage structure

**DOI:** 10.1186/1756-3305-5-168

**Published:** 2012-08-10

**Authors:** Katsuhisa Takumi, Arno Swart, Theo Mank, Erica Lasek-Nesselquist, Marianne Lebbad, Simone M Cacciò, Hein Sprong

**Affiliations:** 1 National Institute of Public Health and Environment (RIVM), Laboratory for Zoonosis and Environmental Microbiology (CIb-LZO), P.O. Box 1, 3720, BA, Bilthoven, The Netherlands; 2 Department of Parasitology, Laboratory of Public Health, Haarlem, The Netherlands; 3 Josephine Bay Paul Center for Comparative Evolution and Molecular Biology, Woods Hole, MA, USA; 4 Department of Diagnostics and Vaccinology, Swedish Institute for Communicable Disease Control, Solna, Sweden; 5 Department of Infectious, Parasitic, and Immunomediated Diseases, Istituto Superiore di Sanita, Rome, Italy

**Keywords:** Giardia lamblia, Giardia intestinalis, Giardia duodenalis, Genetic recombination, Population genetics

## Abstract

**Background:**

*Giardia duodenalis* is a common protozoan parasite of humans and animals. Genetic characterization of single loci indicates the existence of eight groups called assemblages, which differ in their host distribution. Molecular analyses challenged the idea that *G. duodenalis* is a strictly clonal diplomonad by providing evidence of recombination within and between assemblages. Particularly, inter-assemblage recombination events would complicate the interpretation of multi-locus genotyping data from field isolates: where is a host infected with multiple *Giardia* genotypes or with a single, recombined *Giardia* genotype.

**Methods:**

Population genetic analyses on the single and multiple-locus level on an extensive dataset of *G. duodenalis* isolates from humans and animals were performed.

**Results:**

Our analyses indicate that recombination between isolates from different assemblages are apparently very rare or absent in the natural population of *Giardia duodenalis.* At the multi-locus level, our statistical analyses are more congruent with clonal reproduction and can equally well be explained with the presence of multiple *G. duodenalis* genotypes within one field isolate.

**Conclusions:**

We conclude that recombination between *G. duodenalis* assemblages is either very rare or absent. Recombination between genotypes from the same assemblage and genetic exchange between the nuclei of a single cyst needs further investigation.

## Background

*Giardia duodenalis* (syn. *G. lambliaG. intestinalis*) is the etiological agent of giardiasis, a gastrointestinal infection of humans, companion animals, livestock and wildlife. Symptoms of a *G. duodenalis* infection range from asymptomatic to severe diarrhea as well as chronic disease [1]. *G. duodenalis* has a simple life cycle comprising rapidly multiplying, non-invasive trophozoites on the mucosal surface of the intestine, and the production of environmentally resistant cysts that are shed with the host faces. Infectious cysts are transmitted by the faecal-oral route either by direct contact or by ingestion of contaminated food or water [2]. G. *duodenalis* is considered as a species complex, whose members show little variation in their morphology, yet can be assigned to eight distinct assemblages (A to H) based on enzyme electrophoretic and genetic studies [3,4]. Assemblages A and B can infect and multiply in humans and are also found in a wide range of mammals. The remaining assemblages show more restricted host ranges: C and D are predominantly found in canids, E in livestock, F in cats, G in rodents and H in marine vertebrates (seal and gull) [5,6]. In endemic areas where humans and animals live closely together, transmission from human to animals or *vice versa* may occur [7-9]. Direct evidence for transmission from animals to human is lacking, because *Giardia* cysts are shed into the environment, making it very difficult to determine the primary source of the infection. Genetic characterization has been extensively used to assess the role of animals in the epidemiology of human infection and to develop tools for tracing sources of infection. However, the zoonotic potential of *G. duodenalis* remains a major and unresolved issue [1,10,11].

Many molecular epidemiological studies have been based on the analysis of a single marker, often from a limited number of isolates. Using single locus approaches, the zoonotic potential of *G. duodenalis* assemblage A and B appears to be high: Irrespective of the genetic marker used, sequences from human and animal field isolates frequently appeared similar, if not identical [12]. In order to increase the accuracy of genotyping of *G. duodenalis* isolates, multi-locus sequence typing strategies were introduced [13-18]. When genotypes from field isolates were defined using a multi-locus sequence typing scheme, only 2 from the 84 multi-locus genotypes (MLG) of assemblage A and none MLGs (n = 99) of assemblage B appear to have a zoonotic potential [12]. Surprisingly, the genotypes of *Giardia* field isolates repeatedly constituted a combination of loci derived from different assemblages [12]. The latter finding can be explained by two biological phenomena.

One explanation is that a *Giardia* field isolate is not a singular clone, but consists of a mixture of different *Giardia* genotypes. A *Giardia* field isolate is often not more than a DNA extract, obtained either directly from a stool sample or indirectly after (immune-) isolation of faecal cysts. For this situation, the uptake of genetically different *Giardia* cysts from the environment by a host, or subsequent infection of an already infected host, likely without overt symptoms, with a different *Giardia* genotype, must occur. As starting *in vitro* cultures from field samples prove to be very difficult due to variations in excitation and adaptation, and the ever-present bacterial and fungal contamination, it is hard to affirm the clonality of *Giardia* field isolates [19,20]. Alternatively, the *Giardia* isolates are clonal, but the mixing of loci from different assemblages have arisen by (para) sexual recombination, i.e. genetic exchange, between *G. duodenalis* assemblages. Although *G. duodenalis* shows no cytological evidence of meiotic and sexual recombination, several studies challenged the idea that *G. duodenalis* is a strictly clonal organism [21,22]. These studies have demonstrated: (i) the presence in the *G. duodenalis* genome of true homologs of genes involved in meiosis in other eukaryotes [23,24]; (ii) the exchange of genetic material in different chromosomal regions among human isolates of the parasite [25,26]; (iii) the fusion between cyst nuclei (karyogamy) and the transfer of genetic material (episomal plasmids) between them [27]. These results are pivotal for the existence of sexual recombination. Recombination may take place at three levels within *G. duodenalis*: (i) between the two nuclei at an individual level, (ii) between individuals of the same assemblage and (iii) between individuals of different assemblages.

Here, we only address the latter situation, i.e. recombination between assemblages, as this mostly complicates the interpretation of the molecular epidemiological data. Genetic exchange between different assemblages may occur in laboratory cultures or in nature, but it remains to be determined to what extent this occurs in natural populations [28]. Genetic exchange between isolates of different assemblages was addressed using two approaches: The detection of mosaic sequences in three loci and the performance of several tests for clonal reproduction at the population level.

## Methods

### Data collection

A European network of public and veterinary health Institutions that focused on zoonotic protozoan parasites (the ZOOnotic Protozoa Network, ZOOPNET) was established [12]. Within this consortium, a molecular epidemiological database was built, and currently contains information on 3351 *Giardia* isolates, which encompass 4954 sequences from 5 different loci. Although GenBank sequences constitute approximately 45% of the database, limited epidemiological data (mainly country and source of isolation) are available for those isolates. All molecular epidemiological data were stored and analyzed in Bionumerics (Version 6.10; Applied Math, Belgium). A selection of these sequences was made using the same strategy as previously described [4] . For example, sequences that were too short to cover regions of variation within any given assemblage were used only for analysis at the level of that assemblage, but not at the level of sub-assemblage. In addition, when multiple, identical sequences from any given isolate were deposited in GenBank, only the longest available sequence was retrieved.

### DNA-sequence analysis

The loci beta-giardin (BG), glutamate dehydrogenase (GDH), and triose phosphate isomerase (TPI) were sorted into their different genes, assemblages, and sub-assemblages (AI, AII and AIII) as well as alignments along the gene using previously defined references [12]. Tests for intra-assemblage recombination events were performed essentially as described [26]. The four-gamete test, MaxChi, and the four additional tests offered in RDP V3.41, RDP, GENCONV, Chimera, and SiScan [29] were performed. All tests rely on the premise that recombination will produce mosaic sequences. Because the region and length of GDH sequenced varied widely among the isolates, two GDH genealogies were produced; a GDH1, which used sequences that spanned nucleotides 186–1,159 and a GDH2, which used sequences that spanned nucleotides 258–647 where nucleotide positions correspond to the complete sequence of GDH from the WB:C6 genome [30]. Potential recombinants identified by these algorithms, were discarded if both parental sequences were from the same assemblage (potential intra-assemblage recombination).

Sequences were aligned by using MAFFT [31], distance-based analyses were conducted by using Kimura 2-parameter distance estimates, and trees were constructed by using the Neighbor-Joining algorithm, implemented in the Bionumerics program. Bootstrap proportions were calculated by the analysis of 500 replicates for neighbor-joining trees.

### Probability of observing no offspring of recombinant types in the sample

Occurrence of a recombinant of B assemblage and E assemblage at TPI locus of a human isolate is an evidence of genetic exchange between the two assemblages. Under the hypothesis of selective neutrality, we calculated the probability of our observation; each recombinant type was found in only one sample at the particular locus, to statistically test the hypothesis, we made an assumption that a recombinant and a non-recombinant sequence type are selectively neutral, and calculated the probability that each recombinant type was observed exactly once in the samples of DNA sequences under the assumption of selective neutrality. The probability was calculated using the formula [32],

(1) $$\frac{n!}{k!\left|s\left(n,k\right)\right|n_{1}n_{2}\ldots n_{k}}$$

where the symbol *n* is the number of samples at a given locus, the symbol *k* is the number of distinct recombinant types and a non-recombinant type, the function *s(n,k)* is the stirling numbers of the first kind, and the bracket |.| indicates an absolute value.

### Criteria for clonality: Identical genotypes (test d)

The extent to which the predominant genotype was overrepresented can be quantitatively evaluated by calculating the probability *P* of observing as many or more individuals of the particular genotype as actually observed in the sample,

(2) $$P=\sum_{i=m}^{n}\frac{n!x^{i}{\left(1-x\right)}^{n-i}}{i!\left(n-i\right)!}\text{,}$$

where *x* = probability of the multi-locus genotype under the null hypothesis of full recombination, estimated by multiplying the observed frequency of the single-locus genotypes; *n* = number of individuals sampled; and *m* = number of individuals in the sample with the particular genotype [28].

### Criteria for clonality: Absence of recombinant genotypes (test e)

This criterion is the probability of observing as few different multi-locus types as actually observed, given the size of the samples and expected multi-locus type frequencies under the hypothesis of full recombination [28]. The probability was calculated by a Monte Carlo approach by drawing ten-thousand random samples from the multinomial distribution with the vector of expected frequencies in Table 1. The probability was defined to be the fraction of random samples for which the number of distinct multi-locus types are less than or equal to the number of multi-locus types actually observed.

**Table 1:** Frequencies of three-locus types under the hypothesis of free recombination

**Host**	**Human (n = 369)**			**Livestock (n = 45)**		
Assemblage*	**Num**	**Obs.%**	**Exp.%**	**Num**	**Obs.%**	**Exp.%**
000	140	38	6	11	34	2
001	4	**1**	10	0	0	5
010	1	0	10	0	0	5
011	6	**2**	15	0	0	14
100	0	0	9	0	0	5
101	0	0	14	0	0	14
110	1	0	14	0	0	14
111	217	59	22	34	64	43
**Host**	**Cat (n = 17)**			**Dog (n = 55)**		
Assemblage*	**Num**	**Obs.%**	**Exp.%**	**Num**	**Obs.%**	**Exp.%**
000	7	41	7	24	44	11
001	0	0	10	1	**2**	11
010	0	0	10	0	0	12
011	0	0	14	0	0	11
100	0	0	10	1	**2**	14
101	0	0	14	1	**2**	13
110	0	0	14	3	**5**	14
111	10	59	20	25	45	14

* Letter 0 is a wildcard that designates either A (human, livestock, and cat assemblages) or C (dog). Similarly, letter 1 designates one of B (human), E (livestock), D (dog), or F (cat). Expected (Exp.) frequencies were calculated by multiplying the frequencies of genotype 0 at each locus with the frequencies of genotype 1 at each locus in eight possible ways. For comparison, the frequency of observed (Obs.) is displayed as well.

### Criteria for clonality: Linkage disequilibrium (test f)

This is a standard test for non random association between loci, under the assumptions of random mating, and non-overlapping generations [33]. In our database of *G. duodenalis*, two genotypes were recorded at each locus. Hence we refer to one genotype by a letter 0 and to the other genotype by a letter 1. Linkage disequilibrium *D*_*12*_ between locus 1 and locus 2 was calculated using the equation [33],

(3) $$D_{12}=p_{00}-p_{*0}p_{0*}\text{,}$$

where *p*_00_ is the frequency of congruent assemblages 00, *p*_0*_ is the frequency of assemblage whose locus 1 is the type 0 and locus 2 is either 0 or 1, and p_*0_ is the frequency of assemblage whose locus 2 is the type 0 and locus 1 is either 0 or 1.

In the case of three-locus typing, linkage disequilibrium was calculated using the equation 1 in [34],

(4) $$D=p_{000}-p_{0**}p_{*0*}p_{**0}-p_{0**}D_{23}-p_{*0*}D_{13}-p_{**0}D_{12}\text{,}$$

where *D*_*23*_*D*_*13*_ and *D*_*12*_ are pair-wise linkage disequilibrium between the three loci 1, 2, and 3, *p*_000_ is the frequency of multi-locus types 000. The symbol * is a wildcard for either genotype 0 or 1. Thus, *p*_00*_ is the frequency of the assemblages having genotype 0 at the first and at the second loci and genotype at the third locus is either 0 or 1. *P*-values for linkage disequilibrium were obtained by a Monte Carlo approach as described in the test *e* but using *D* as the test statistic.

### Index of association

Index of association for *G. duodenalis* was calculated using the observed frequencies of two genotypes at each of the three loci (Table 1) using the equation 3 in [35]. Variance of the index of association was calculated using the equation 4 in [35].

## Results

### Frequency of inter-assemblage recombination events within three loci

In a recent study, recombination between homologous loci from different assemblages was identified in at least three *G. duodenalis* isolates [26]. In this study, a limited number of sequences from two loci (a selection from GenBank entries until 2008) were analyzed. We performed the same approaches [26] to analyze the 4582 sequences from three loci stored in our database. Here, only potential recombinant events where parental sequences could be identified were taken into account. In the 1633 sequences from the BG locus, no inter-assemblage recombination events were detected. From the 1325 TPI sequences, only one isolate (D6) displayed a recombination event between assemblages B and E. In the GDH sequences (n = 1624) of two isolates, K2521 and R24, potential inter-assemblage recombination events occurred (Table 2, Additional file 1: Table S1). Both recombination events were identified previously. One 551-bp GDH sequence (GQ337967) in GenBank displayed a recombination event, where the last ~95 bp did not align with any other *G.duodenalis,* but were similar to *H. sapiens* RIO kinase 2 (AK225348) [36]. No inter-assemblage recombination was identified in the GDH sequences of the K4016 and SweCat17 isolates. These two were identified previously as potential recombinants [26], but turned out to be identical to other members of sub-assemblage AIII [13].

**Table 2:** Isolates with potential inter-assemblage recombination within loci

**Source (Isolate ID)**	**Locus**	**Mixed**	**Reference**	**Nested-PCR**	**Description**
Cattle (K2521)	GDH	A/E	DQ182604 [37]	Yes	18SRDNA sequence of this rectal faecal sample was identified as Ass E. Other study samples contained Ass AI, AIII or Ass E.
Surface water (R24)	GDH	A/B	EU350516 [38]	Yes	Environmental water sample,which contained very few cysts.
Human (D6)	TPI	B/E	EU272164 [39]	Yes	Faecal sample derived from rural community in Egypt, where people commonly live in close contact with their livestock.
Cattle (K4016)	GDH	Is Ass AIII	DQ182607 [37]	Yes	AIII is excluded as recombinant from AI and AII as they latter two could not be identified as parental strains.
Cat (Swecat171)	GDH	Is Ass AIII	EU769223 [14]	Yes	

DNA sequences presented in this table were all generated by a (semi)-nested PCR for GDH [40] and TPI [41]. All these isolates were identified previously as potential recombinants [26].

**Figure 1:**
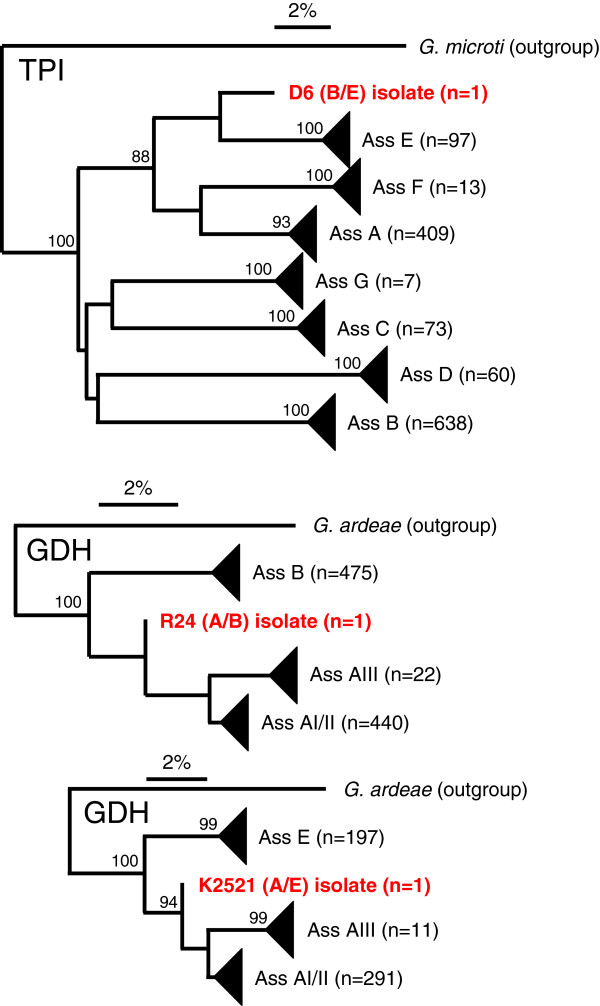
**Phylogenetic analysis based on single gene sequences of TPI and GDH, as obtained using neighbour joining.** Bootstrap values > 85 are indicated. Terminal branches were collapsed for clarity. The three recombinant sequences (D6, R24 and K2521) form singular branches between the existing assemblage structures.

Lack of inter-assemblage recombination will eventually result in the independent and divergent evolution of alleles from different assemblages [42]. Phylogenetic analyses of the available sequences of GDH and TPI generally display divergent and independent evolution (not shown, but see [4,12]). The sequences of the three isolates which underwent potential recombination events (Table 2) turned out to be exceptional, as they do not cluster significantly with any of the other sequences from the same locus (Figure 1). We conjectured that a successful recombinant is (at least) equally fit as its parents, resulting in offspring. To statistically test this hypothesis, we made an assumption that a recombinant and a non-recombinant type are selectively neutral in producing offspring, and calculated the probability that each recombinant type in the sample was observed exactly once in the sample. For GDH locus, we counted two recombinant types each occurring exactly once in the sample and counted the rest of 1622 non-recombinant types. The probability of this observation under the assumption of selective neutrality was estimated to be 0.5%. For TPI locus, we counted one recombinant type occurring exactly once in the sample and counted the rest of 1324 non-recombinant types. The probability of this observation under the assumption of selective neutrality was 6% (see Methods for calculation of the probability).

### Frequency of inter-assemblage recombination events between loci of two assemblages

Absence of recombination within the three loci is insufficient to rule out inter-assemblage recombination, as these loci are relatively conserved regions and have an average size of only ~ 500 base pairs. The probability of detecting inter-assemblage recombination becomes significantly higher when multiple, independent loci on different chromosomes are used. Potential recombination events were assessed between pairs of assemblages, which occur most frequently together, i.e. which share similar host categories. Recombination between assemblages A and B was studied in *G. duodenalis* isolates from humans, between assemblages C and D in dog isolates, between A and F in cat isolates and between A and E in isolates from livestock. The presence of a particular multi-locus type in great excess is often the most robust and significant evidence of clonal reproduction, i.e. lack of recombination [28]. Here, a multi-locus type of a *G. duodenalis* isolate was based on the concatenated assemblage-typing of the three different loci BG, GDH and TPI. Only two multi-locus types are found in great excess in humans, namely AAA (38%) and BBB (59%). Of the six possible combinations of A and B in three-locus genotypes, only 3% are found (Table 1). The same was observed in the three other host categories. Two multi-locus types are found in great excess: AAA and EEE in livestock, AAA and FFF in cats, and CCC and DDD in dogs (Table 1). The contrast to the *observed* frequencies of three-locus genotypes is clear when the *expected* frequencies of three-locus types under the hypothesis of free recombination is calculated (Table 1), supporting the assertion that sexual reproduction between assemblages in *G. duodenalis* did not leave its trace on the population structure.

Three formal tests for clonality were performed and all three yielded highly significant results in the four *G. duodenalis* populations isolated from human, livestock, cat, and dog (Table 3). These results support that clonal reproduction had indeed shaped the observed population structures, independent of the origins from which the genetic materials of *G. duodenalis* were isolated. A fourth statistical test, Index of association (I_A_), was originally developed to index the extent of clonality within bacterial populations [35]. Small estimates for the variance indicate that the index values were significantly different from zero, and hence that recombination has been rare or absent.

**Table 3:** Tests for clonality applied to the three-locus typing datasets

**Criterion**	**Human**	**Livestock**	**Cat**	**Dog**
Identical genotypes widespread (*d*)	<10^-9^	10^-6^	10^-4^	10^-9^
Absence of recombinant genotypes (*e*)	<10^-4^	<10^-4^	<10^-4^	<10^-4^
Linkage disequilibrium (*f*)	0.38*	0.24*	0.38*	0.42*
Index of association (I_A_)	1.8 ± 0.00	2.0 ± 0.02	2.0 ± 0.04	1.5 ± 0.02

* P value < 10^-4^. Italic letters in parentheses corresponds to the same tests in [43]. I_A_-values which are significantly different from zero indicates that recombination has been rare or absent.

## Discussion

Occurrence of one recombination event between assemblage B and assemblage E at the TPI locus of a human isolate is an indication that genetic exchange between the two assemblages has occurred. However, detection of a single recombinant is important but not sufficient information to assert the nature of recombination between *Giardia duodenalis* assemblages. In fact, in a considerable number of isolates present in the database, this recombinant was found only once. The test under the assumption of selective neutrality between a recombinant and a non-recombinant type supported that a *Giardia* recombinant might contribute genetically little to the population structure of *Giardia duodenalis*. It could be argued that a zoonotic transmission event between a human individual and domestic livestock is unlikely to produce a novel viable *G. duodenalis* assemblage by a recombination of existing assemblages at TPI locus. Molecular typing of the *Giardia* isolates at GDH locus in addition indicated that a recombinant at the GDH locus contributes genetically very little to the population structure of *Giardia duodenalis*. The observation of a recombination event between a GDH DNA-sequence (GQ337967) and a DNA sequence probably derived from its host (sheep) is highly anomalous. A PCR-artifact or sequencing error seems more likely.

The lack of observation of selectively neutral or fitter recombinant types was not due to a limited capacity of detecting a recombination event that could be associated with a single locus typing method. The analysis of the three-locus sequence typing data by applying a battery of population genetic tests supported that the contribution of recombination events between the three loci to the population structure of *G. duodenalis* is also limited. These population genetic tests have been applied to a number of microorganisms, to support a clonal population structure of *Leishmania* as well as *Trypanosoma*, and to reject a clonal population structure of *Candida albicans*[28]. Judging by the weight of the evidence provided by the population genetic analyses of the molecular typing datasets of *G. duodenalis*, both a single and multi-locus based typing method, we opt for the hypothesis that the population structure of *G. duodenalis* assemblages is clonal. This conclusion is by no means in conflict with the existence of recombination within the two nuclei of a cyst at the genomic and the cellular level [21,22], nor does our analysis exclude the possibility of intra-assemblage recombination.

Our molecular epidemiological database consists of 1312 human and animal isolates with two or more loci. Fifteen percent (n = 197) of these isolates constitute a combination of loci derived from different assemblages. Based on the analyses of this study, we conclude that these isolates are a mixture of different *Giardia* genotypes from different assemblages. This is probably an underestimation, as mixed infections are not always detected. Unequal loads or stability of the microorganisms of interest in the original sample, preferential immuno-purification or preferential binding of primer pairs to one of microorganisms will result in the detection of only one of them. PCR assays based on the use of assemblage-specific primers have been developed to show the frequency of mixed infections [8,44]. It also is possible that another fraction of the field-isolates in this database consists of a mixture of different *Giardia* genotypes from the *same* assemblage. Besides allelic sequence heterogeneity, the latter may be an additional explanation for the presence of ambiguous nucleotides (“double peaks”) present in the sequences of many isolates. Unfortunately, it is still not possible to distinguish between a clonal isolate and an isolate consisting of a mixture of the same assemblage. A PCR-based approach using a generic set of primers appears to be unreliable for the detection of mixed infections.

## Conclusion

Molecular epidemiology is probably the best method available to study transmission dynamics of *G. duodenalis* in and between human and animal populations. The relatively poor genetic resolution of the available single loci used for these studies could not be solved by combining several of these loci to a multi-locus genotyping scheme. Multilocus-sequence typing directly on field isolates is hampered by the occurrence of mixed infections in both animals and humans. Our data imply that genetic recombination between genotypes of different assemblages is either very rare or absent in the *G. duodenalis* population. Thus, multilocus sequence types from field isolates, which consist of more than one assemblage, should be interpreted as a mixed infection. Improved *in vitro* culturing methods and novel molecular typing methods, such as single cyst PCR, are required to investigate the directionality and frequency of animal to human transmission and to determine the frequency of intra-assemblage recombination events [45].

## Competing interest

The authors declare that they have no competing interests.

## Authors’ contributions

TM, ML, SC, and HS performed data collection and sequence analysis. KT and AS performed population genetic and mathematical analysis. HS performed phylogenetic analysis. KT, SC, EL and HS were involved in the study design and interpretation of the results. KT and HS drafted the manuscript and wrote the final version. All authors read and approved the final manuscript.

## Supplementary Material

Additional file 1 **Table S1.** Inter-assemblage recombination events within three loci. Click here for file
