# Supplementary material for: Population-based analyses of Giardia duodenalis is consistent with the clonal assemblage structure
Source: Parasit Vectors. 2012 Aug 10;5:168. doi: 10.1186/1756-3305-5-168 (PMC3431248; doi:10.1186/1756-3305-5-168)
Supplement: Additional file 1 — Table S1. Inter-assemblage recombination events within three loci. [file 1756-3305-5-168-S1.doc]

Supplementary Table I: ***Inter-assemblage recombination events within three loci***

**EU272164**

**DQ182603**

**EU350516**

The last ~ 95 bp of **GQ337967** do not align with *giardia* GDH present in the db. The last 95 bp are more similar to Homo sapiens RIO kinase 2 (AK225348)
